# Cytotoxic and Apoptotic Effects of Govaniadine Isolated from* Corydalis govaniana* Wall. Roots on Human Breast Cancer (MCF-7) Cells

**DOI:** 10.1155/2018/3171348

**Published:** 2018-07-24

**Authors:** Nivethika Sivakumaran, Sameera R. Samarakoon, Achyut Adhikari, Meran K. Ediriweera, Kamani H. Tennekoon, Neelika Malavige, Ira Thabrew, Ram Lal (Swagat) Shrestha

**Affiliations:** ^1^Institute of Biochemistry, Molecular Biology and Biotechnology, University of Colombo, 90 Cumaratunga Munidasa Mawatha, Colombo, Sri Lanka; ^2^Central Department of Chemistry, Tribhuvan University, Kirtipur, Kathmandu, Nepal; ^3^Department of Microbiology, Faculty of Medical Sciences, University of Sri Jayewardenepura, Gangodawila, Nugegoda, Sri Lanka; ^4^Department of Chemistry, Tribhuvan University, Amrit Science Campus, Kathmandu, Nepal

## Abstract

Current breast cancer therapies have limitations in terms of increased drug resistance resulting in short-term efficacy, thus demanding the discovery of new therapeutic agents. In this study, cytotoxic activity and apoptotic effects of govaniadine isolated from* Corydalis govaniana* Wall. roots were determined on human breast cancer (MCF-7) cells. The SRB assay result revealed that govaniadine led to dose- and time-dependent cytotoxic effect in MCF-7 cells along with less cytotoxicity against MCF-10A cells. Govaniadine-induced apoptosis was also accompanied by upregulation of* Bax*,* p53*, and* Survivin* mRNA expression as assessed by real time PCR analysis. Flow cytometric analysis with Annexin V and PI staining indicated that govaniadine is a potent inducer of apoptosis in MCF-7 cell lines. Distinctive morphological changes contributed to apoptosis and DNA laddering were observed in govaniadine-treated MCF-7 cells. Caspase-7 was significantly activated in treated MCF-7 cells. Govaniadine-treated MCF-7 cells also showed enhanced levels of intracellular reactive oxygen species (ROS) and glutathione S-transferase (GST) and decreased levels of glutathione (GSH). The results indicate that govaniadine has potent and selective cytotoxic effects against MCF-7 cells and the potential to induce caspase 7 dependent apoptosis in MCF-7 cells by activation of pathways that lead to oxidative stress.

## 1. Introduction

Breast cancer is the most common cancer in women worldwide, resulting in 350,000 deaths each year [1]. The potential of using natural products as anticancer agents was recognized in the 1950s by the US National Cancer Institute (NCI), and more than 60% of current therapies for cancer are derived from natural sources, including plants [2, 3]. Unfortunately, current therapies for breast cancer are often limited by short-term efficacy due to the nonspecific targeting, high toxicity to normal tissues, undesirable side effects, and drug resistance. Therefore, novel drugs with fewer side effects, greater therapeutic efficiency, and low cost are needed to treat breast cancer [4]. Inhibition of apoptosis is associated with cancer; thus apoptosis is a popular target in the development of novel anticancer drugs. MCF-7 cells lack caspase-3, which is one of the main initiators of apoptotic pathways; thus they become highly resistant to apoptosis and develop resistance against most chemotherapeutic drugs within a few months to a few years [5, 6].

**Figure 1:**
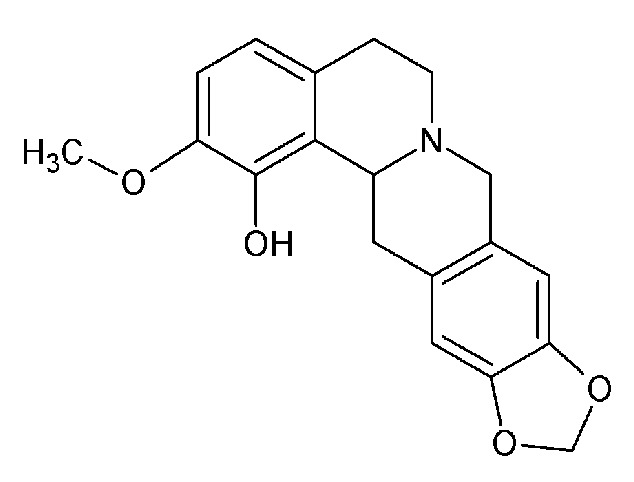
Structure of govaniadine.

*Corydalis govaniana* Wall. is a glabrous herb distributed in the Himalayas of Nepal, Pakistan, and India. It grows in damp and shady places at 2400–4800 m altitude [7]. Ethnomedically, the roots have been used in the treatment of syphilis, scrofula, cutaneous infections, diarrhea, and dysentery [8, 9]. Plant extracts, pure compounds, and alkaloids from different species of this genus have been effective against hepatitis, cirrhosis, ascites, amoebiasis, liver cancer, and other tumors [10]. They also caused sedation and improved immunological function. The excellent activity profile of the genus* Corydalis *and ethnobotanical uses of this plant led to conducting an isolation of a new compound govaniadine (Figure 1) along with three known tetrahydroprotoberberine-type alkaloids [11]. Govaniadine was shown to possess some bioactivities such as urease inhibition activity and analgesic activity [11, 12]. In addition, it was shown to possess pharmacokinetic properties such as* in vitro* metabolism and plasma protein binding [13], but its anticancer activity has not yet been studied. Therefore, in the present study* in vitro *cytotoxic activity and apoptotic effects of govaniadine on MCF-7 breast cancer cells were evaluated.

## 2. Methods

### 2.1. Compound Govaniadine

Govaniadine was isolated from the plant* Corydalis govaniana* Wall., a plant which is endemic to China, as well as the Himalayas of Nepal, Pakistan, and India, and also found in mountainous regions of Eastern Africa [11]. Chloroform extract obtained, after solvent partitioning of the methanol extract of the whole plant was used to isolate pure govaniadine. For the isolation, chloroform extract was separated in a silica gel column with acetone and hexane as the mobile phase. Structure of govaniadine was elucidated with the help of ^1^H NMR, ^13^C NMR, 2D NMR techniques (COSY, HSQC, and HMBC), HR-EIMS, UV, and IR spectroscopy. The molecular formula of govaniadine was confirmed by HRESI-MS which displayed pseudomolecular ion peak at [M+H]^+^ ion at m/z 326.1383 (calcd. for C_19_H_19_O_4_ + H = 326.1392)] [11].

### 2.2. Cell Culture and Reagents

Human breast cancer cell line [MCF-7, ER^+^ (ATCC, HTB-22TM)] was cultured in Dulbecco's Modified Eagle Medium (DMEM) (Invitrogen, Carlsbad, CA, USA) supplemented with 10% Fetal bovine serum (FBS), 100 U/mL of penicillin, 0.1 mg/mL streptomycin, and 0.01 mg/mL insulin. Normal mammary epithelial cell line [MCF-10A (ATCC® CRL-10317)] was grown in Mammary Epithelium Basal Medium (MEBM) (Lonza, Walkersville, MD, USA). Both MCF-7 and MCF-10A cells were maintained in a humidified incubator at 37°C with 5% CO_2_. All the cell lines and 10% FBS were purchased from the American type cell culture (ATCC), Rockville, MD, USA. All chemicals were purchased from Sigma-Aldrich (St. Louis, MO, USA) unless otherwise specified.

### 2.3. *In Vitro* Cytotoxicity Assay

MCF-7 and MCF-10A cells were trypsinized using 25% v/v trypsin/EDTA, plated in cell culture treated 96-well plates (5 x 10^3^ cells/ well) and incubated for 24 h. After incubation, cells were treated with govaniadine (1, 2, 4, 8, or 16 *μ*M) and maintained at 37°C with CO_2_ for 24, 48, or 72 h. After the incubation period, Sulforhodamine B (SRB) assay was performed as described previously [14] to assess cell viability. Absorbance was read at optical density (OD) 540 nm (Synergy™ HT, BioTek, USA) and percentage cell viability was calculated. Negative controls contained only 0.1% DMSO and medium whereas paclitaxel was used as the positive control [15].

### 2.4. Determination of Cell Morphology Using Light Microscopy

To determine the effect of govaniadine on the morphology of MCF-7 cells, cells (2x10^5^ cells/mL) maintained in DMEM for 24 h were exposed to different concentrations (1, 2, 4, 8, and 16 *μ*M) of govaniadine for further 24, 48, or 72 h at 37°C in 5% CO_2_. Control cells were treated with 0.1% DMSO. After the incubation period, morphological changes of the cells were observed and photographed on an inverted phase contrast microscope (Olympus CKX41SF, Japan).

### 2.5. Determination of Cell Morphology and Nuclear Changes Using Acridine Orange/Ethidium Bromide (AO/EB) and Hoechst Staining

MCF-7 cells harvested by trypsinization upon reaching 70-80% confluence were seeded (5x10^4^ cells/well) on cell culture treated cover slips placed in a 24-well cell culture plate and exposed to different concentrations (2, 4, 8, and 16 *μ*M) of govaniadine for 24 h at 37°C in 5% CO_2_. Control cells were treated with 0.1% DMSO. After 24 h, cells were fixed with 4% formaldehyde, and AO/EB solution (10 *μ*L) and Hoechst stain (0.005 mg/mL, 10 *μ*L) were added for 2–5 min. Cells were then observed under a fluorescence microscope (Olympus Co., Tokyo, BX51TRF, Japan).

### 2.6. Measurement of Caspase 7 Activity

MCF-7 cells (2 x 10^5^ cells/mL) were seeded in 96-well plates and maintained for 24 h at 37°C in 5% CO_2_. After 24 h, cells were exposed to govaniadine (1 and 2 *μ*M) for further 24 h. Control cells were treated with 0.1% DMSO. Caspase-Glo® 3/7 assay (Promega, Madison, WI, USA) was used to measure caspase 7 activity according to the manufacturer's instructions, and luminescence was measured using a luminescence plate reader (Synergy™ HT, BioTek, USA).

### 2.7. Quantitative Real Time PCR Analysis (RT-qPCR)

Cells were grown in T_25_ flasks (2 x 10^5^ cells/mL) for 24 h at 37°C in 5% CO_2_ and treated with govaniadine (1 and 2 *μ*M) in triplicate and incubated for further 24 h. Control cells were treated with 0.1% DMSO. After 24 h, cells were harvested and total RNA was extracted with TRIzol® Reagent (Invitrogen; Thermo Fisher Scientific, Inc., Carlsbad, CA, USA) according to the manufacturer's protocol. cDNA synthesis and RT-qPCR were performed as described previously [15]. RT-qPCR was performed in Stratagene Mx3000P real time PCR machine using MESA Green qPCR Master Mix Plus for SYBR Assay (Eurogentec, Seraing, Liège, Belgium). Data were normalized to an internal reference gene* GAPDH*, and relative gene expression was assessed using the comparative Ct method (2^−ΔΔCt^) [16]. The sequences of primers used for RT-qPCR are tabulated in Table 1.

### 2.8. Quantitative Determination of Apoptosis by Using Flow Cytometry

MCF-7 cells (2x10^5^ cell/mL) cultured in T_25_ cell culture flasks for 24 h at 37°C in 5% CO_2_ were exposed to govaniadine (4, 8, and 16 *μ*M) for further 24 h. Control cells were treated with 0.1% DMSO. After 24 h, apoptosis mediated by the govaniadine on MCF-7 cells was detected by the Annexin V/ Propidium Iodide (PI) apoptosis detection kit (Santa Cruz, Texas, USA) according to the manufacturer's instructions by using a Partec Cyflow® Cube 6 Flow Cytometer. The data was analyzed with De Novo FCS Express version 4 software, and all experiments to detect apoptosis were done in triplicate.

### 2.9. DNA Fragmentation Analysis

MCF-7 cells (2x10^5^ cell/mL) were treated with 4, 8, and 16 *μ*M doses of govaniadine incubated for 48 or 72 h, respectively. Medium containing 0.1% DMSO and thymoquinone were used as negative and positive controls, respectively. After the incubation period, DNA fragmentation was analyzed as described by [17]. Gel electrophoresis was carried out with extracted DNA and visualized under UV light to assess the effect on DNA fragmentation (Quantum-ST4 1100/20 M; Fisher Biotec Pty Ltd., Wembley, Australia) following electrophoresis on a 2.0% agarose gel stained with ethidium bromide (EB).

### 2.10. Reactive Oxygen Species (ROS) Assay

MCF-7 cells were seeded (2x10^5^ cell/mL) in 96-well plates for 24 h at 37°C in 5% CO_2_. After 24 h, MCF-7 cells were treated with govaniadine (1 and 2 *μ*M) in triplicate and incubated for 24 h. Control cells were treated with 0.1% DMSO. An assay for the intracellular conversion of nitro blue tetrazolium (NBT) to formazan by superoxide anion was used to measure the generation of reactive oxygen species in cells [18]. NTB was subsequently added at a final concentration of 1.2 mM to the wells and incubated in the dark for 1 h at 37°C. The formazan content of the cells was then solubilized with 100 mL of DMSO, and the absorbance was measured at OD 630 nm (Synergy™ HT Multi-Mode Microplate Reader).

### 2.11. Measurement of Intracellular Glutathione (GSH) and Glutathione S-Transferase (GST)

MCF-7 cells were seeded (2x10^5^ cell/mL) in 24-well plates and maintained for 24 h at 37°C in 5% CO_2_. Then the cells were exposed to different concentrations of govaniadine (1 and 2 *μ*M) for further 24 h. Control cells were treated with 0.1% DMSO. After 24 h, cells were trypsinized and pelleted. Cell suspension of approximately 10^6^ cells per mL in an appropriate buffer was prepared and homogenized using sonication in ice. Cell homogenate was transferred to 1.5 mL eppendorf tubes and centrifuged at 15,000 rpm for 10 min at 4°C. The resulting supernatant (cell lysate) was transferred to a new 1.5 mL micro centrifuge tube and kept on ice. Total amount of protein present in the cell lysate was determined using bovine serum albumin (BSA) as the standard. Reduced GSH was estimated as described previously [19]. The activity of GST was determined as described by [20]. Intracellular GSH and GST levels were expressed as *μ*M/mg of cell lysate protein.

### 2.12. Statistical Analysis

GraphPad Prism version 5 (San Diego, California, USA) was used for statistical analyses. Experiments were repeated at least three times, and data are represented as the average and standard deviation (mean ± SD) of three independent experiments. One-way ANOVA with Tukey's posttest was used in caspase 7 analysis and measurement of intracellular ROS, GSH, and GST levels. Two-way ANOVA was used in quantitative real time PCR analysis. One-way and two-way ANOVA analysis were used for comparison between groups and P<0.05 was considered to indicate a statistically significant difference.

## 3. Results

### 3.1. Cytotoxic Effects of Govaniadine

**Figure 2:**
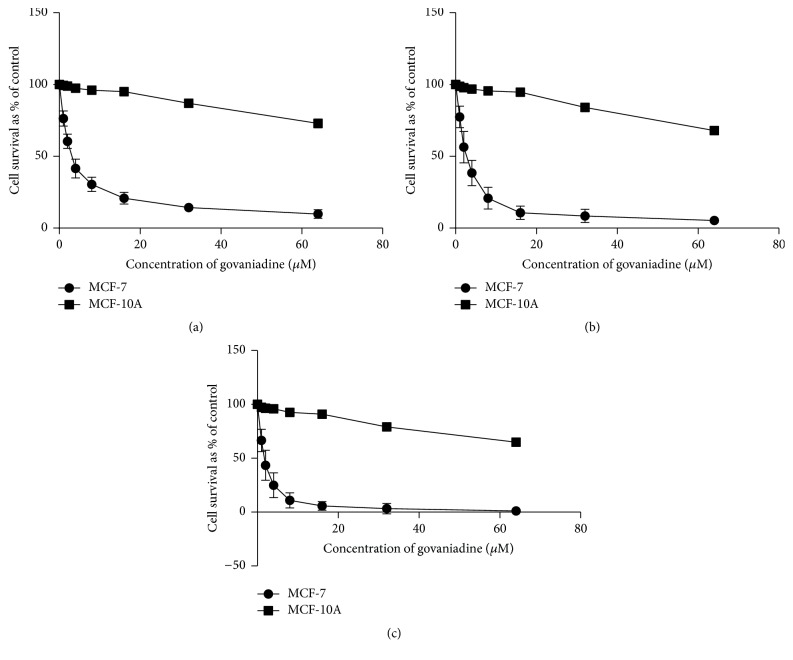
Cytotoxic effects of govaniadine in MCF-7 and MCF-10A cells as determined by the SRB assay. The graphs (a), (b), and (c) show cytotoxic effects of govaniadine in MCF-7 and MCF-10A cell lines for 24, 48, and 72 h postincubation, respectively. Means ± SD values obtained from three different experiments were used to calculate IC_50_ values.

**Figure 3:**
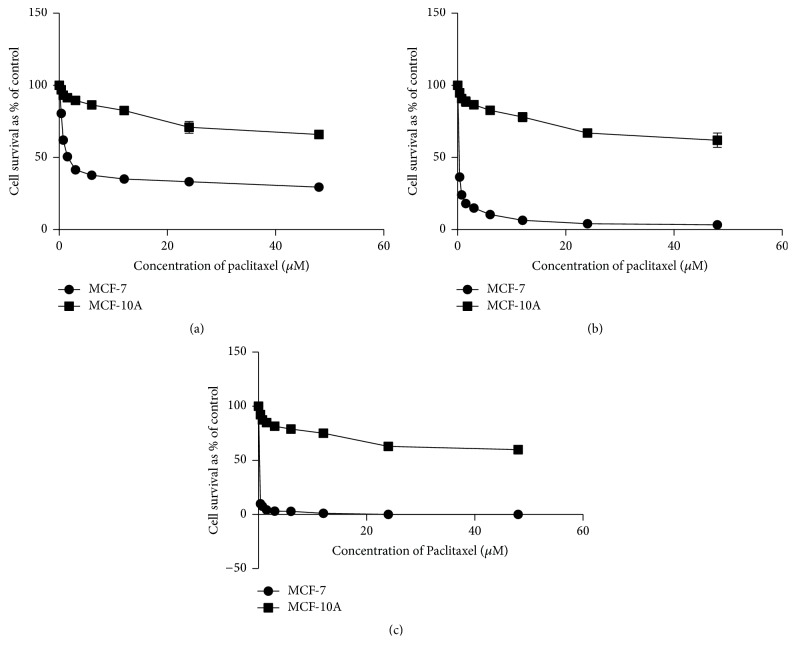
Cytotoxic effect of paclitaxel in MCF-7 and MCF-10A cells as determined by SRB assay. The graphs (a), (b), and (c) show cytotoxic effects of paclitaxel in MCF-7 and MCF-10A cell lines for 24, 48, and 72 h postincubation, respectively. Means ± SD values obtained from 3 different experiments were used to calculate IC_50_ values.

The response of MCF-7 and MCF-10A cells to govaniadine is shown in Figures 2(a), 2(b), and 2(c). The dose of govaniadine causing 50% inhibition (IC_50_) of MCF-7 cells at 24, 48, and 72 h postincubation periods were 3.05±0.27, 2.52±0.31, and 1.6±0.23 *μ*M, respectively, and the IC_50_ values of govaniadine against MCF-10A cells at 24, 48, and 72 h postincubation periods were 269.0±0.25, 223.4±0.32, and 194.9±0.24 *μ*M, respectively. Figures 3(a), 3(b), and 3(c) show the cytotoxic effects of paclitaxel against MCF-7 and normal breast epithelial MCF-10A cells. IC_50_ values for paclitaxel against MCF-7 cells at 24, 48, and 72 h postincubation periods were 2.21±0.15, 0.13±0.05, and 0.0089±0.006 *μ*M, respectively, and against MCF-10A cells 11.92±1.5, 6.057±2.45, and 3.107±1.98 *μ*M, respectively.

### 3.2. Morphological Observations Using Phase Contrast Microscopy

**Figure 4:**
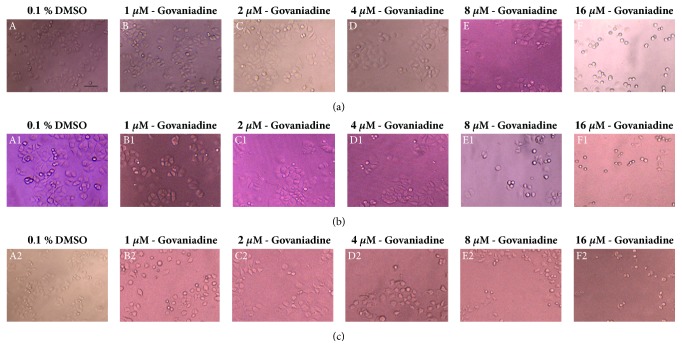
Effects of govaniadine on the morphology of MCF-7 cells after 24 (a), 48 (b), and 72 (c) h of postincubation. A, A1, and A2 control cells (0.1% DMSO); B, B1, and B2 cells treated with 1 *μ*M of govaniadine; C, C1, and C2 cells treated with 2 *μ*M of govaniadine; D, D1, and D2 cells treated with 4 *μ*M of govaniadine; E, E1, and E2 cells treated with 8 *μ*M of govaniadine; F, F1, and F2 cells treated with 16 *μ*M of govaniadine. The cells were examined under an Olympus CKX41SF inverted phase contrast microscope using a 20 X objective lens (magnification 200X). Scale bar: 100 *μ*m.

Incubation of MCF-7 cells with increasing concentrations of govaniadine for 24, 48, and 72 h increased the number of apoptotic cells. Reduction in cell volume, cytoplasm shrinkage, chromatin condensation, and formation of membrane-bound apoptotic bodies were frequently observed. Control cells (Figure 4) retained their normal angular or polygonal shape and spread regularly in the culture plate, while MCF-7 cells treated with low doses of govaniadine (Figure 4) showed loss of the normal monolayer-like growth, and few cells had shrunken cytoplasm, condensed chromatin, and loss of normal shape. MCF-7 cells treated with higher doses of govaniadine (Figure 4) showed marked changes in morphology associated with late stage of apoptosis, such as shrinkage, round shape, and condensed and fragmented chromatin and cytoplasm, to produce apoptotic bodies. Additionally, cell numbers were decreased in a concentration-dependent manner.

### 3.3. Morphological Observations Using Fluorescence Microscopy

**Figure 5:**
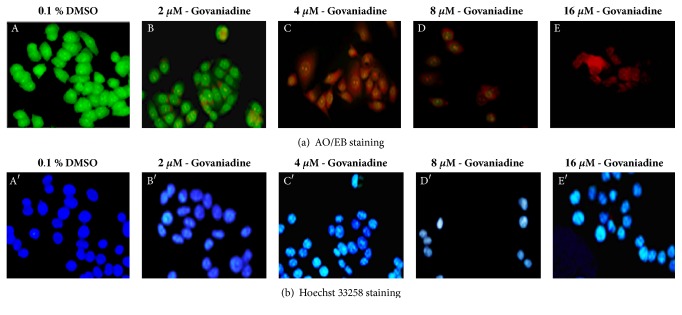
Cell apoptosis observed using fluorescence microscope (200×). Cells were treated with different dose of govaniadine for 24 h and stained with (row (a)) acridine orange-ethidium bromide and (row (b)) Hoechst 33258. (A and A′) untreated cells (0.1% DMSO), (B and B′) cells treated with 2 *μ*M of govaniadine, (C and C′) cells treated with 4 *μ*M of govaniadine, (D and D′) cells treated with 8 *μ*M of govaniadine, and (E and E′) cells treated with 16 *μ*M of govaniadine. Scale bar: 100 *μ*m.

AO/EB and Hoechst 33258 fluorescence staining were used to confirm govaniadine-induced apoptosis in MCF-7 cells. AO/EB staining showed uniform green cells in the control (Figure 5- A), whereas apoptotic cells in the early stage were marked by granular yellow-green (Figure 5- B), and apoptotic cells in the late stage were marked with concentrated and asymmetrically localized orange nuclei (Figure 5: C, D, and E) under fluorescence microscope on exposure to govaniadine. Nuclei with brighter hypercondensed chromatin, strongly bound to fluorescent dyes, were observed in MCF-7 cells treated with govaniadine on Hoechst 33258 staining (Figure 5: B′, C′, D′, and E′). The control cells had intact nuclei with uniformly dispersed chromatin (Figure 5- A′).

### 3.4. Effect of Govaniadine on Caspase 7 Activity

**Figure 6:**
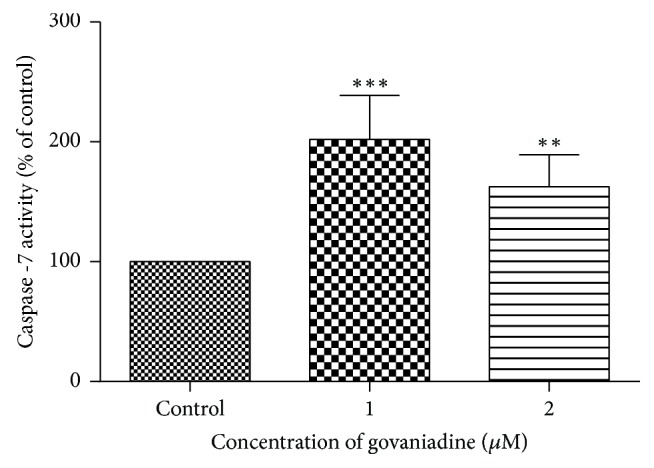
Effect of govaniadine on the caspase-7 activity in MCF-7 cells. MCF-7 cells were treated with 0.1% DMSO (control), 1 *μ*M, and 2 *μ*M of govaniadine, respectively. Mean ± S.D values of three independent experiments carried out in triplicate were used (^*∗∗∗*^P<0.05, ^*∗∗*^P<0.01 when compared to the control).

Caspase-7 activity in response to govaniadine is shown in Figure 6. Caspase-7 was activated significantly (P<0.05 and P<0.01 at 1 *μ*M and 2 *μ*M doses of govaniadine, respectively) when MCF-7 cells were treated with govaniadine. A dose-dependent activation of caspase-7 was observed.

### 3.5. Expression of Apoptosis Related Genes in the Cells Treated with Govaniadine

**Figure 7:**
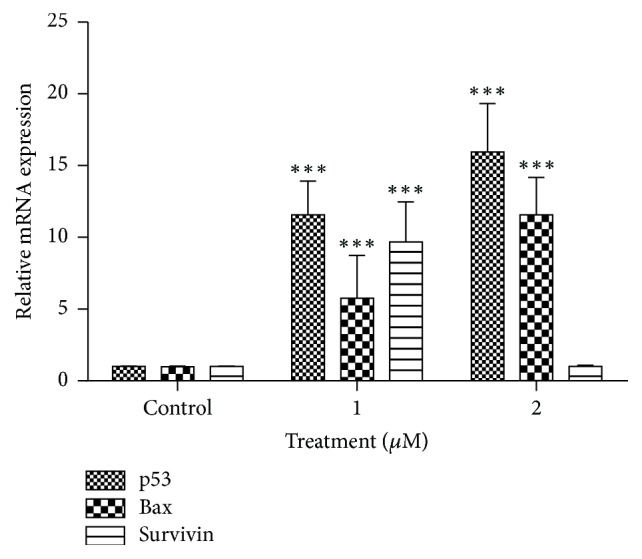
Effect of govaniadine on (a)* p53*, (b)* Bax*, and (c)* Survivin* mRNA expression in MCF-7 cells at 24 h incubation. MCF-7 cells were treated with 0.1% DMSO (control), 1 *μ*M, and 2 *μ*M of govaniadine, respectively. Relative expression was calculated by qRT-PCR using 2  ^∧^  (−ΔΔCT) method. The results are presented as mean ± SD (*n* = 3); ^*∗*^*p* < 0.05 versus control.

Expression level of apoptosis related genes was determined by evaluating mRNA levels of* p53*,* Bax*, and* survivin* using RT-qPCR. Govaniadine-treated MCF-7 cells at 24 h incubation showed (Figure 7) upregulation of tumor suppressor,* p53*, and proapoptotic* Bax* (fold change:* p53*: 11.37 and 15.42 and* Bax*: 5.07 and 11.66 in response to 1 and 2 *μ*M concentrations of govaniadine, respectively). However, the expression of* survivin *gene was not regulated at the highest dose (2 *μ*M) of govaniadine while the cells treated with 1 *μ*M caused a 9.08-fold increase of mRNA levels (Figure 7).

### 3.6. Determination of Govaniadine-Induced Apoptosis by Flow Cytometry

**Figure 8:**
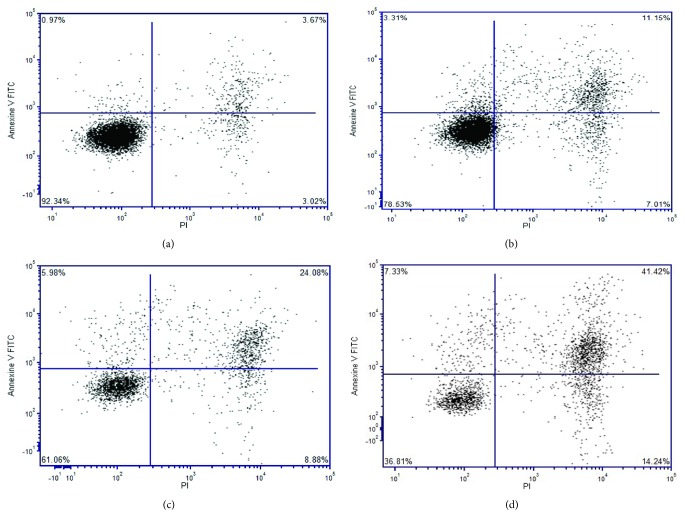
Induction of apoptosis in MCF-7 cells by govaniadine determined by Annexin V-PI flow cytometry technique. (a) Control (0.1% DMSO), (b), (c), and (d) treated with govaniadine 4, 8, and 16 *μ*M, respectively. The lower left quadrant represents intact viable cells (Annexin-FITC and PI negative). The upper left quadrant represents early apoptotic cells (Annexin-FITC positive and PI negative). The upper right region represents late apoptotic cells (Annexin-FITC negative and PI positive). The lower left quadrant represents necrotic cells (Annexin-FITC and PI positive). The data are presented as dot plots of Annexin V/FITC against PI of at least three independent tests. Data were analyzed by specific software, FCS Express 5.

Induction of apoptosis by govaniadine was quantitatively determined by flow cytometric analysis using Annexin V-FITC and PI fluorescence staining kit. The percentage of early apoptotic cells increased in a dose-dependent manner (Figure 8). The early apoptotic cells increased from 3.31% at 4 *μ*M to 7.33% at 16 *μ*M of govaniadine compared to 0.97% in the control at 24 h postincubation. The number of late apoptotic cells increased to 11.15% at 4 *μ*M of govaniadine and increased to 41.42% at 16 *μ*M of govaniadine.

### 3.7. DNA Fragmentation Analysis

**Figure 9:**
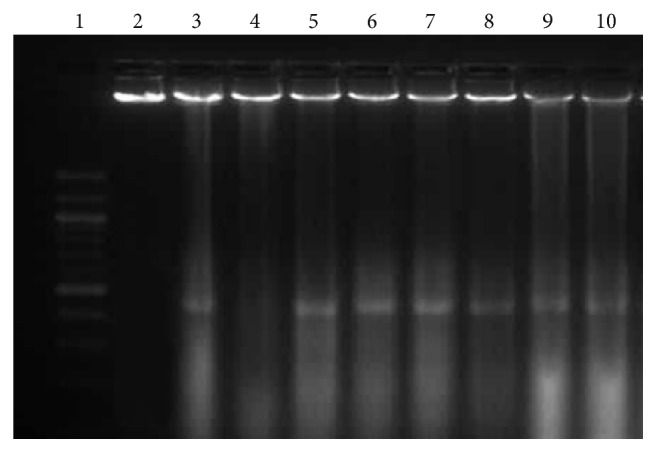
DNA fragmentation analysis. Gel electrophoresis was carried out with DNA extracted from MCF-7 cells, after treatment with govaniadine. Lane 1: 100 bp size marker, Lane 2: control (0.1% DMSO), lane 3: 60 *μ*M thymoquinone (positive control for fragmentation analysis) for 48 h, lane 4: 60 *μ*M thymoquinone for 72 h, lane 5: 4 *μ*M govaniadine for 48 h, lane 6: 8 *μ*M govaniadine for 48 h, lane 7: 16 *μ*M govaniadine for 48 h, lane 8: 4 *μ*M govaniadine for 72 h, lane 9: 8 *μ*M govaniadine for 72 h, lane 10: 16 *μ*M govaniadine for 72 h.

DNA fragmentation is a hallmark feature of apoptosis. MCF-7 (Figure 9) cells treated with govaniadine showed a DNA fragmentation in a time- and dose-dependent manner with both high molecular weight DNA and smaller DNA fragments extending from 2 kbp to 100 bp. The control treated with 0.1% DMSO showed clear bands of intact DNA.

### 3.8. Intracellular ROS, Glutathione, and Glutathione S-Transferase Levels

**Figure 10:**
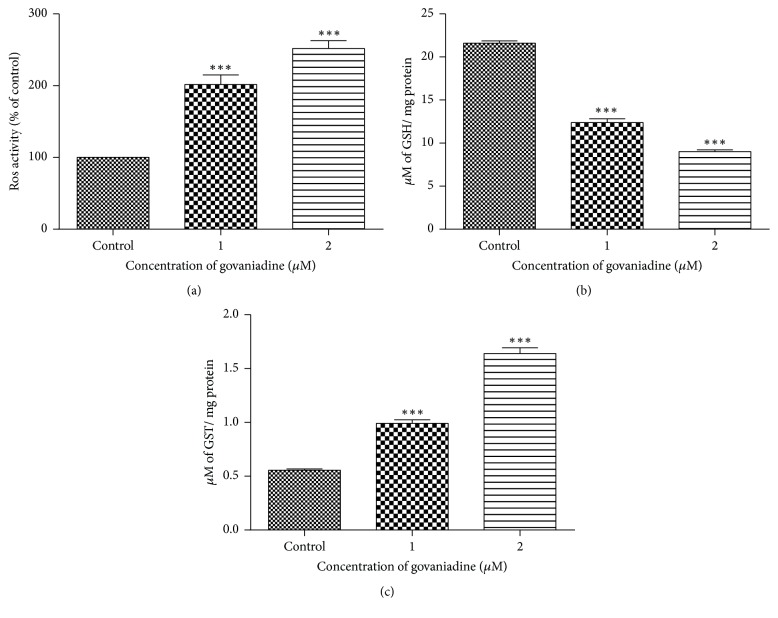
Effects of govaniadine on (a) ROS production, (b) GSH level, and (c) GST level in MCF-7 cells. Values were presented as mean ± S.D (n=3) of three independent experiments carried out in triplicate (^*∗∗∗*^P<0.05 when compared to the control).

As shown in Figure 10(a), ROS production was at the basal level in control DMSO-treated MCF-7 cells. In contrast, treatment with govaniadine for 24 h resulted in dose-dependent significant (p<0.05) increase of ROS production (Figure 10(a)). Intracellular GSH levels decreased significantly (p<0.05) in the cells treated with 1 and 2 *μ*M in a dose-dependent manner (Figure 10(b)). In addition, GST level was increased significantly (p<0.05) on govaniadine treatment (Figure 10(c)).

## 4. Discussion

Previous studies on medicinal plants commonly used by the inhabitants in China, India, and western Himalaya revealed that* Corydalis govaniana* Wall. is the most important species based on its traditional use [21, 22]. Different parts of this plant and isolated phytochemicals have been used for the prevention and treatment of various health ailments for many decades [8, 9, 21, 22]. Previous studies on* C. govaniana *led to the isolation of many tetrahydroberberine alkaloids [9, 11, 22, 23]. Although several tetrahydroprotoberberine-type alkaloids have been reported from* C. govaniana *Wall. plant, anticancer activity of pure isolated compounds has not yet been reported. Therefore, in the present study,* in vitro *cytotoxic activity and apoptotic effects of the govaniadine on MCF-7 breast cancer cells were evaluated.

*In vitro *cytotoxic activity and apoptotic effects of govaniadine were preliminary investigated by assessing the cell viability and cell morphology of govaniadine-treated MCF-7 cells. Our data demonstrated that govaniadine exerted significant cytotoxic effects against MCF-7 breast cancer cells by inducing marked morphological changes associated with apoptosis and a decrease in the number of cells in a time- and dose-dependent manner. Anticancer drugs that are capable of inducing selective apoptosis of cancer cells, with minimal side effects on normal cells, are highly desirable for therapeutic purposes [24, 25]. Cytotoxicity evaluation against normal mammary epithelial cells demonstrated that govaniadine possesses less cytotoxic effects on MCF-10A cells. Concentrations of govaniadine which were cytotoxic to the human breast cancer MCF7 cells did not exhibit the inhibition in MCF-10A cells. “Potent cytotoxic effects in MCF-7 cells and low cytotoxic activity in MCF-10A cells as compared to the reference drug paclitaxel suggest that govaniadine is more selective towards MCF-7 cells than the paclitaxel. In addition, IC_50_ values of commercially available anticancer agents such as tamoxifen and doxorubicin in MCF-7 and MCF-10A cell lines reported by other authors [26] suggest that govaniadine's cytotoxicity towards MCF-7 cells is more selective than tamoxifen and doxorubicin. Several naturally derived alkaloids with prospective anticancer properties against MCF-7 cells, such as berberine, evodiamine, and piperine, have already been reported by other authors [27] and the reported IC_50_ values of above mentioned alkaloid compounds [28–30] suggest that govaniadine exhibits considerably higher inhibition than above mentioned alkaloids in MCF-7 cells”.

In view of the morphological features of apoptosis observed in response to govaniadine, the proapoptotic effects of govaniadine in MCF-7 cells were further clarified by investigating caspase-7 activation and enzymatic cleavage of DNA into oligonucleosomal fragments. Apoptosis involves the sequential activation of a cascade of proteases, known as caspases. There are two classes of caspase, initiators and effectors, and the effector caspases include caspase-3 and -7 that exhibit differential activity towards multiple substrate proteins [31].

Caspase-3 is commonly activated by numerous death signals and cleaves a variety of important cellular proteins [32]. It is responsible for DNA fragmentation and some of the distinct morphological features of apoptotic cells such as membrane blebbing and formation of apoptotic bodies. In MCF-7 cells, caspase-3 is not expressed as a result of a 47-base-pair deletion within exon 3 of the* casp-3* gene; thus only caspase-7 mediates apoptosis [33]. In the present study apoptosis induced by changes in cell morphology and DNA fragmentation is therefore independent of caspase-3. Other authors have reported that caspase 3 independent apoptosis is mediated by other effector caspases such as caspase-6 or -7 [34, 35]. In the present study, we used thymoquinone rather than paclitaxel as the positive control for assessing DNA fragmentation as the latter is known to induce apoptosis in MCF7 cells without DNA fragmentation [36]. Thymoquinone has been reported to cause apoptotic cell death in MCF-7 cells as long term treatment [37].

p53 is a crucial tumor suppressor that functions as a transcription activator of a panoply of target genes essential for cell cycle arrest, DNA repair, and apoptosis. p53 transcriptionally activates proapoptotic* Bax* and represses antiapoptotic* survivin *gene expression [38–40]. Evaluation of* p53, Bax, *and* survivin *expression is a common approach used for analysis of apoptosis in response to anticancer compounds [41]. Upregulation of* p53 *and* Bax *and downregulation of* survivin* have been already reported in MCF-7 in response to other compounds such as berberine, zerumbone, and taxol [42, 43]. Govaniadine increased expression of* p53 *and* Bax* mRNA in MCF-7 cells in a dose-dependent manner at 24 h. However, the expression of* survivin* significantly increased in MCF-7 cells at the lowest dose (1 *μ*M) with no considerable change in surviving expression at the highest (2 *μ*M) dose used in the present study. Increased expression of* survivin *upon treatment with paclitaxel and adriamycin (clinically used anticancer drugs) and inhibition of survivin phosphorylation on Thr^34^ expression have been reported to cause decrease in* survivin* expression in MCF-7 cells as reported in a study carried out by Wall et al., 2003 [44]. Hence, survivin phosphorylation on Thr^34^ independently by the cyclin-dependent kinase of p53 regulation may be one of the reasons for the upregulation of* survivin *in response to low (1 *μ*M) dose of govaniadine [44]. However, further investigations are needed to confirm the effect of govaniadine on the regulation of* survivin* gene expression.

Induction of apoptosis by govaniadine was further confirmed by flow cytometric analysis. One of the hallmarks of apoptosis is the externalization of phospholipid phosphatidylserine (PS) by translocation from the inner to outer layer of plasma membrane for recognition by phagocytes during early stage of apoptosis [45]. Hence, phosphatidylserine can serve as specific target for the detection of early apoptotic cells. Annexin V-FITC which has high binding affinity for phosphatidylserine is an appropriate conjugate for identification of early stage apoptosis. Simultaneously, PI is a nucleic acid binding red-fluorescent dye which is impermeant to live cells and early apoptotic cells, but it stains late apoptotic and necrotic cells with red fluorescence, binding tightly to the nucleic acids in the cell [46]. Percentage of early and late apoptotic cells increased when MCF-7 cells were treated with different concentrations of govaniadine.

It is well known that apoptosis is induced either by depletion of endogenous antioxidants or by generation of free radicals [47]. Cells are known to thrive in low levels of reactive oxygen species (ROS), but a relative increase in ROS induces cell cycle arrest and apoptosis. ROS-modulating drugs are, however, being proposed as therapeutic strategies to selectively target the destruction of cancer cells [48]. The results of our study indicate that the govaniadine induces a dose-dependent increase in ROS production at 24 h, suggesting MCF-7 cell line is sensitive to govaniadine with regard to its oxidative stress-induced cytotoxicity. In the present study, GSH levels were significantly lower and GST levels were significantly increased in treated cells when compared to untreated cells. Cellular GSH plays an important role in protection against oxidative stress-induced injury [49]. Depletion of GSH levels has been shown to enhance susceptibility to oxidative stress-induced cytotoxicity [50]. GSTs are involved in catalyzing the GSH conjugate formation [50]. Reduction of GSH level and concomitant increase of GST activity in response to govaniadine observed in the present study are likely to have further potentiated cytotoxic and apoptotic effects of govaniadine on MCF-7 cells.

In summary, we suggest that govaniadine demonstrates selective inhibition of MCF-7 breast cancer cells through reactive oxygen species (oxidative stress) mediated apoptosis. Furthermore, govaniadine causes upregulation of* Bax* and* p53* and consequent expression of caspase 3 in MCF-7, which suggests activation of intrinsic pathway of apoptosis. These findings will help to give a proper understanding on the action of govaniadine in future studies.

## 5. Conclusions

Taken together, the results of the present study clearly show that the govaniadine is able to induce cytotoxicity, apoptosis, and oxidative stress-induced cell death in MCF-7 breast cancer cells. Therefore, govaniadine might be a leading candidate in the development of a chemotherapeutic agent for treating estrogen receptor positive breast cancer. However, the molecular mechanistic aspects of its effect are not fully identified. Thus, further research work is needed to establish the detailed anticancer mechanism of govaniadine.

## Figures and Tables

**Table 1:** Primers used for real time PCR.

Gene	**Forward Primer (5**′**-3**′**)**	**Reverse Primer (5**′**-3**′**)**

Bax	TCCAGGATCGAGCAGGGCGAA	CGATGCGCTTGAGACACTCGCT

p53	TCTGGCCCCTCCTCAGCATCTT	TTGGGCAGTGCTCGCTTAGTGC

GAPDH	GGCATTGCCCTCAACGACCAC	ACATGACAAGGTGCGGCTCCCTA

Survivin	TGGCCGCTCCTCCCTCAGAAAA	GCTGCTGCCTCCAAAGAAAGCG

## Data Availability

The data and materials supporting the conclusions of this article are included within the article.
